# High risk of gastrointestinal hemorrhage in patients with systemic sclerosis

**DOI:** 10.1186/s13075-019-2078-5

**Published:** 2019-12-26

**Authors:** Yi-Ting Lin, Yun-Shiuan Chuang, Jiunn-Wei Wang, Ping-Hsun Wu

**Affiliations:** 1 Department of Family Medicine, Kaohsiung Medical University Hospital, Kaohsiung, Taiwan; 2 Graduate Institute of Clinical Medicine, College of Medicine, Kaohsiung Medical University, Kaohsiung, Taiwan; 3 School of Medicine, College of Medicine, Kaohsiung Medical University, Kaohsiung, Taiwan; 4 Division of Gastroenterology, Department of Internal Medicine, Kaohsiung Medical University Hospital, Kaohsiung, Taiwan; 5 Division of Nephrology, Department of Internal Medicine, Kaohsiung Medical University Hospital, 100 Shih-Chuan 1st Road, Kaohsiung, 807 Taiwan

**Keywords:** Systemic sclerosis, Gastrointestinal bleeding, Population-based study, Taiwan national health insurance research database

## Abstract

**Background:**

Systemic sclerosis (SSc), a life-threatening autoimmune disease characterized by vasculopathy. Numerous SSc patients demonstrate gastrointestinal (GI) involvement but the delicate GI bleeding risk remains sparse. We aimed to explore the role of SSc in determining the long-term risk of GI bleeding, including bleedings of upper (peptic and non-peptic ulcers) and lower GI tracts.

**Methods:**

Patients with SSc diagnosis were identified from the Catastrophic Illness Patient Database and the National Health Insurance Research Database from 1998 to 2007. Each SSc patient was matched with five SSc-free individuals by age, sex, and index date. All individuals (case = 3665, control = 18,325) were followed until the appearance of a GI bleeding event, death, or end of 2008. A subdistribution hazards model was assessed to evaluate the GI bleeding risk with adjustments for age, sex, and time-dependent covariates, comorbidity, and medications.

**Results:**

The incidence rate ratios of GI bleeding were 2.38 (95% confidence interval [CI], 2.02–2.79), 2.06 (95% CI, 1.68–2.53), and 3.16 (95% CI, 2.53–3.96) for over-all, upper, and lower GI bleeding events in SSc patients. In the competing death risk in the subdistribution hazards model with time-covariate adjustment, SSc was an independent risk factor for over-all GI bleeding events (subdistribution hazard ratio [sHR] 2.98, 95% CI, 2.21–4.02), upper GI bleeding events (sHR 2.80, 95% CI, 1.92–4.08), and lower GI bleeding events (sHR 3.93, 95% CI, 2.52–6.13).

**Conclusion:**

SSc patients exhibited a significantly higher risk of over-all and different subtype GI bleeding events compared with the SSc-free population. The prevention strategy is needed for these high GI bleeding risk groups.

## Introduction

Systemic sclerosis (SSc) is a connective tissue disease characterized by immune dysregulation with excessive collagen and extracellular matrix deposition and alterations in microvasculature; it leads to fibrosis of the skin and internal organs, most frequently the heart, lungs, gastrointestinal (GI) tract, and kidney [1, 2]. SSc is rare but can have poor prognosis and low survival rate. Its prevalence is 56.3–341 cases per million population [3, 4], but the incidence and prevalence of SSc have increased, particularly in the USA and Australia [5].

GI involvement is one of the most common disease manifestations in SSc. According to EULAR Scleroderma Trial and Research (EUSTAR) cohort, 3% SSc patients died related to gastrointestinal causes as the primary cause of death [6]. Videocapsule endoscopy in patients with SSc demonstrated a high frequency of GI mucosal abnormalities, with a marked predominance of vascular mucosal damage [7]. The involvement of the GI tract may lead to profuse hemorrhage. A hospital-based study demonstrated higher risks of severe GI hemorrhage in patients with SSc [8]. However, small sample size, cross-sectional study design without longitudinal follow-up, and lack of comparison groups limit the result interpretation.

To address the paucity of knowledge regarding the natural history of GI bleeding in patients with SSc, we conducted a nationwide database study by analyzing a comprehensive national database over a 10-year period. Our primary aim was to evaluate the long-term risk of GI bleeding, including bleedings of upper (peptic and non-peptic ulcers) and lower GI tracts, in SSc patients compared to the general population.

## Methods

### Data source

This study was based on a Longitudinal Health Insurance Database, the National Health Insurance Research Database (NHIRD), maintained by the Taiwan National Health Research Institute. Taiwan launched a compulsory social insurance program, the National Health Insurance (NHI) program, to provide health care for all the island’s residents since 1995. The NHI program had enrolled more than 23 million Taiwanese residents and more 99% annual coverage rate currently [9, 10]. It covers all medical benefit claims of ambulatory and inpatient care and has been extensively used for many epidemiological studies. The NHIRD established a registry system for “catastrophic illnesses,” including cancer, end-stage renal disease, and several autoimmune diseases. Both outpatient and inpatient claims of beneficiaries with a catastrophic illness certificate are collected in the catastrophic illness profile. The Bureau of NHI performs routine validation of the diagnoses by reviewing the original medical charts of all patients applying for catastrophic illness registration [10]. In this study, all cases of SSc patients were obtained from the Registry of Catastrophic Illness Database. The database includes all relevant information on the catastrophic illness certificate status, such as diagnostic codes in the format of International Classification of Disease, Ninth Revision (ICD-9), date of diagnosis, date of death, date of every clinic visit, details of prescriptions, expenditure amounts, and outpatient and inpatient claims data of the beneficiaries with catastrophic illnesses. This study was approved by the Research Ethics Board of Kaohsiung Medical University Hospital (KMUHIRB-EXEMPT(I)-20150041).

### Study design and population

**Fig. 1:**
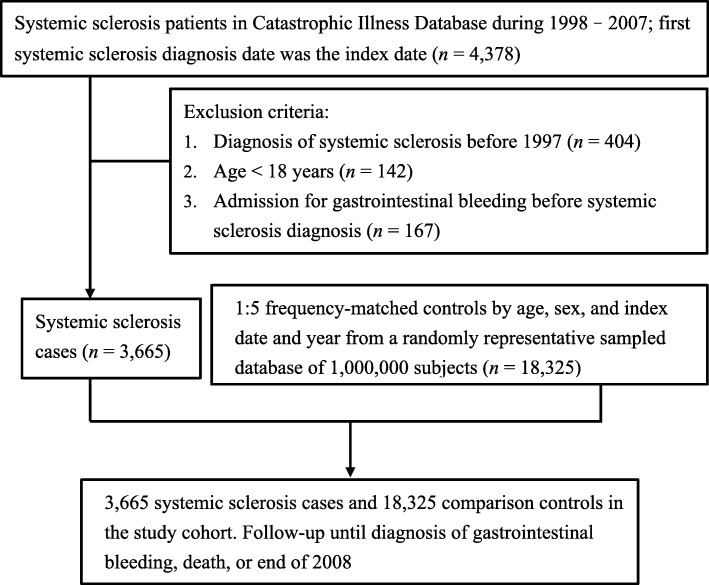
Flow of participant recruitment in the Taiwan National Health Insurance. Systemic sclerosis patients in Catastrophic Illness Database during 1998–2007; first systemic sclerosis diagnosis date was the index date (*n* = 4378). Nonsystemic sclerosis patients were enrolled from the Longitudinal Health Insurance Database (LHID) 2005, which excluded all systemic sclerosis diagnoses (ICD-9 CM 710.1). LHID is a representative database comprising 1,000,000 subjects from the entire National Health Insurance enrollees randomly sampled by the National Health Research Institute of Taiwan

A diagnosis of SSc (ICD-9 codes 710.1) was defined according to the American College of Rheumatology (ACR) diagnostic criteria and verified using the Registry for Catastrophic Illness Patient Database. Patients with newly identified SSc between January 1, 1998, and December 31, 2007, were selected as the SSc cohort. The date on which a patient diagnosed as SSc for the first time was defined as the index date. We excluded individuals diagnosed as having SSc before 1997 (*n* = 404), those younger than 18 years (*n* = 142), and those admitted due to GI bleeding before SSc diagnosis (*n* = 167). A comparison cohort was randomly selected from the Longitudinal Health Insurance Database, which comprises 1,000,000 randomly selected people from the NHI beneficiaries. We matched each patient with SSc with five non-SSc controls according to age, sex, and index date. We enrolled 3665 SSc patients and 18,325 non-SSc controls in the study cohort. The follow-up duration was until diagnosis of GI bleeding, death, withdrawal from the insurance program, or the end of 2008 (Fig. 1).

### SSc diagnostic criteria

For the verification of SSc diagnosis, one rheumatologist is required to provide relevant clinical and laboratory information for the NHI review, and the review committee of the Registry of Catastrophic Illness assesses applications according to the criteria of the ACR for SSc. The ACR proposed the following classification criteria for SSc: the major criterion was proximal scleroderma and the minor criteria were sclerodactyly, digital pitting, scarring or loss of substance on the finger pads, and bibasilar pulmonary fibrosis. Patients fulfilling the major criterion or at least two minor criteria can be diagnosed as having SSc [11].

### Definition of GI bleeding and comorbidities

GI bleeding was defined as hospitalization with a major diagnosis of GI bleeding after the index date during the study period (Additional file 1: Table S1). The GI bleeding endpoint was further categorized as upper and lower GI bleeding [12, 13] (Additional file 1: Table S1). Peptic and non-peptic ulcer bleeding were defined on the basis of ICD-9-CM codes reported previously [14–17] (Additional file 1: Table S1). Peptic ulcer bleeding was also proven through endoscopic examination and was associated with endoscopic therapy (epinephrine injection, heat-probe thermocoagulation, and hemoclipping) for ulcer bleeding and use of proton pump inhibitors (PPIs).

The comorbidities identified in this study were diabetes mellitus, hypertension, dyslipidemia, chronic kidney disease (CKD), chronic obstructive pulmonary disease (COPD), coronary artery disease (CAD), and uncomplicated peptic ulcer disease, which were defined by disease codes based on at least two ambulatory visits or hospitalizations for a principal diagnosis during 1 year before index date (Additional file 1: Table S1). In addition, we retrieved information on the bleeding risk medications, including antiplatelets, anticoagulants, traditional nonsteroidal anti-inflammatory drugs (NSAIDs), cyclooxygenase-2 (COX-2) inhibitors, and steroids. Moreover, anti-acid secretory drug use, including PPIs and histamine type-2 receptor antagonists (H2RA), was also considered (Additional file 1: Table S2). Medications were identified and classified on the basis of the National Drug Classification System and the Anatomic Therapeutic Chemical Code, which is an internationally accepted drug classification system coordinated by the World Health Organization Collaborating Centre for Drug Statistics Methodology. Concomitant medication users were defined as those using medications for > 28 days during the observation period.

### Statistical analysis

All data are expressed as frequencies (percentages) or means ± standard deviations. Student’s *t* test was used to assess parametric continuous data between groups, and the chi-squared test was used to assess categorical data. The occurrence of GI bleeding events in the SSc and comparison groups has a competing risk for death; therefore, the differences between the proportions of individuals that developed GI bleeding in the SSc and control groups during the 10-year follow-up period were analyzed using the cumulative incidence competing risk (CICR) method [18] with a modified log-rank test.

To consider the risk of death expected among subjects, subdistribution hazards were derived from competing risk regression using Fine and Gray hazards model [19]. The interaction between the predictors and event time was noted, and variables that did not fulfill the assumption or were deemed to be time-dependent were entered as continuous time-dependent covariates. Moreover, a time-dependent covariate multivariate subdistribution hazard model was performed to analyze independent risk factors for GI bleeding. Because death prior to the development of GI bleeding events was considered a competing risk event, we applied the proportional hazards model for the subdistribution of a competing risk to estimate the subdistribution hazard ratios (sHRs) and 95% confidence intervals (CIs) in relation to the primary outcomes [20]. Analyses were performed using the SAS (version 9.4; SAS Institute Inc., Cary, NC, USA) and Stata (version 15, College Station, TX, USA). All statistical tests were two-tailed. A *p* of < 0.05 was considered statistically significant.

## Results

### Subject characteristics

The demographic characteristics of the study subjects are listed in Table 1. Both cohorts comprised 20.7% men (760 in the SSc group and 3800 in the control group). The SSc group had a higher proportion of comorbidities such as diabetes mellitus, hypertension, dyslipidemia, CKD, COPD, CAD; a history of uncomplicated peptic ulcer disease; and concurrent use of antiplatelets, warfarin, traditional NSAIDs, COX-2 inhibitors, PPIs, H2RAs, and steroids (*p* < 0.001).

**Table 1:** Clinical characteristics of systemic sclerosis patients and age- and sex-matched controls

	Case (*n* = 3665)	Control (*n* = 18,325)	
	*N* (%)	*N* (%)	*p* value
Age, years	49.1 ± 14.9	49.1 ± 14.9	1
Sex			
Male	760 (20.7)	3800 (20.7)	1
Comorbidity			
Diabetes mellitus	374 (10.4)	1090 (5.9)	< 0.001
Hypertension	723 (20.0)	2116 (11.5)	< 0.001
Dyslipidemia	424 (11.7)	829 (4.5)	< 0.001
Coronary artery disease	339 (9.4)	770 (4.2)	< 0.001
Chronic kidney disease	121 (3.3)	116 (0.6)	< 0.001
Chronic obstructive pulmonary disease	461 (12.8)	916 (5.0)	< 0.001
History of uncomplicated peptic ulcer disease	262 (7.1)	644 (3.5)	< 0.001
Medications			
Antiplatelets	491 (13.4)	705 (3.8)	< 0.001
Warfarin	45 (1.2)	43 (0.2)	< 0.001
Traditional nonsteroidal anti-inflammatory drugs	1250 (34.1)	3015 (16.5)	< 0.001
Cyclooxygenase-2 inhibitors	485 (13.2)	100 (0.5)	< 0.001
Proton pump inhibitors	155 (4.2)	103 (0.6)	< 0.001
Histamine type 2 receptor antagonist	101 (2.8)	233 (1.3)	< 0.001
Steroids	1370 (37.4)	564 (3.1)	< 0.001

### Risk of GI bleeding in SSc patients and non-SSc controls

During the 10-year follow-up period (mean follow-up, 5.34 and 5.81 years for the SSc and control groups, respectively), 191 cases (5.21%) in the SSc group and 675 cases (3.68%) in the control group experienced GI bleeding, corresponding to an incidence rate of 976.37 (95% CI, 847.27–1125.14) and 410.76 (95% CI, 380.91–442.95) per 100,000 person-years, respectively. GI bleeding risk was significantly higher in SSc patients than in non-SSc controls, with an incidence rate ratio of 2.38 (95% CI, 2.02–2.79, *p* < 0.001; Table 2). The incidence rates of upper and lower GI bleeding were higher in SSc patients, with an incidence rate ratio of 2.06 (95% CI, 1.68–2.53, *p* < 0.001) for upper GI bleeding and 3.16 (95% CI, 2.53–3.96, *p* < 0.001) for lower GI bleeding (Table 2). Among upper GI bleeding events, the non-peptic ulcer bleeding events were more common than peptic ulcer bleeding events in SSc patients (incidence rate ratio, 3.00; 95% CI, 2.39–3.76; *p* < 0.001; Table 2).

**Table 2:** Incident rates of gastrointestinal bleeding among systemic sclerosis and control groups

Systemic sclerosis (*n* = 3665)			Controls (*n* = 18,325)		
Clinical outcome	No.	Incidence rate^a^ (95% CI)	No.	Incidence rate^a^ (95% CI)	Incidence rate ratio (95% CI)
All gastrointestinal bleeding	191	976.37 (847.27–1125.137)	675	410.76 (380.91–442.95)	2.38 (2.02–2.79)
Upper gastrointestinal bleeding	117	591.75 (493.68–709.30)	473	286.89 (262.17–313.94)	2.06 (1.68–2.53)
Peptic ulcer bleeding	84	422.96 (341.53–523.81)	393	238.01 (215.60–262.74)	1.78 (1.40–2.25)
Non-peptic ulcer bleeding	101	507.83 (417.85–617.19)	281	169.46 (150.76–190.48)	3.00 (2.39–3.76)
Lower gastrointestinal bleeding	105	528.20 (436.24–639.53)	277	167.06 (148.50–187.94)	3.16 (2.53–3.96)

^a^Incidence of gastrointestinal bleeding: per 100,000 person-years

**Fig. 2:**
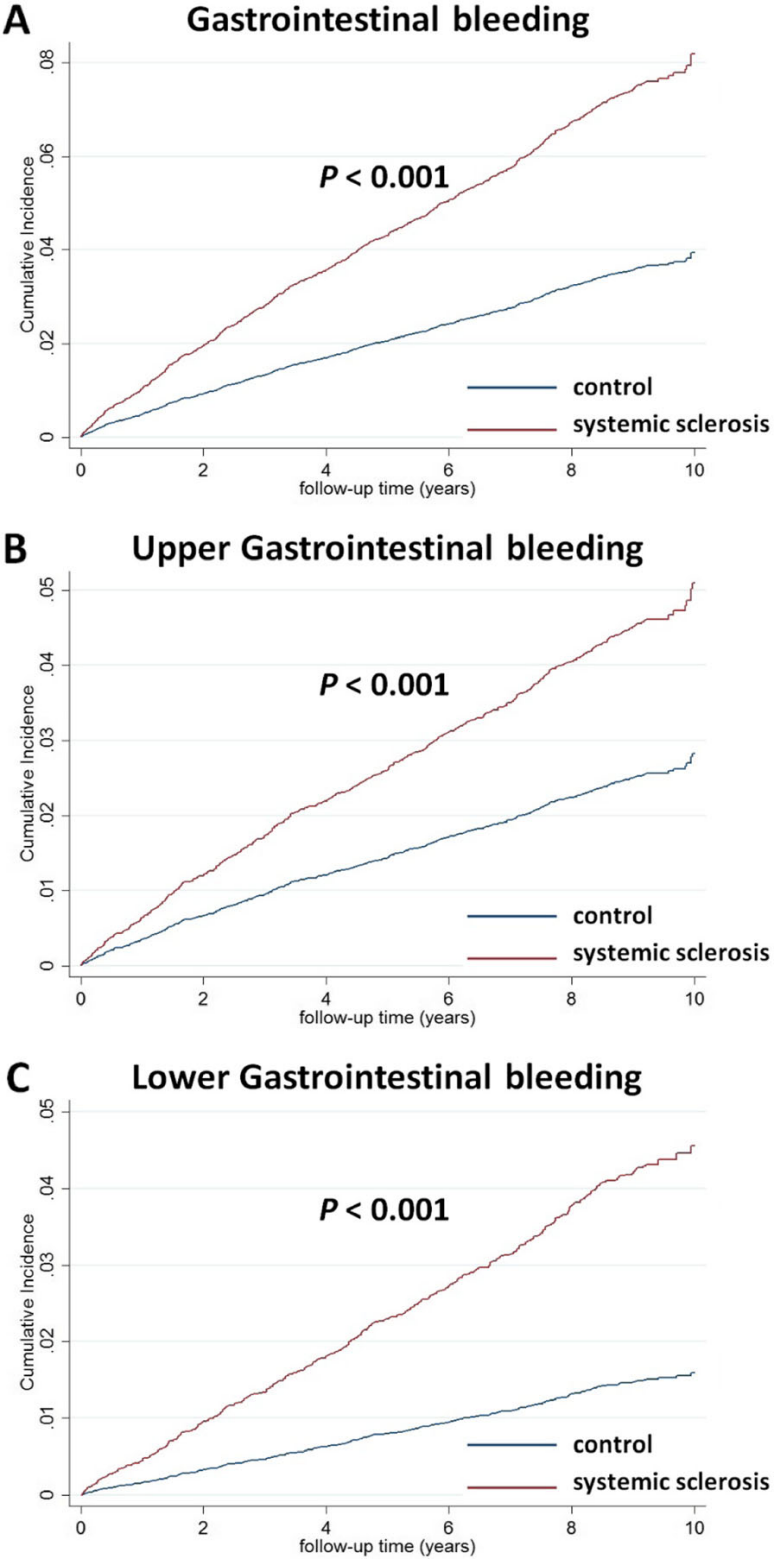
Cumulative incidences of **a** gastrointestinal bleeding (modified log-rank, *p* < 0.001), **b** upper gastrointestinal bleeding (modified log-rank, *p* < 0.001), and **c** lower gastrointestinal bleeding (modified log-rank, *p* < 0.001) estimated of cumulative incidence competing risk method in patients with and without systemic sclerosis

In CICR analysis with death considered as a competing cause of risk, the cumulative incidences of GI bleeding were higher in SSc patients than in matched controls (modified log-rank, *p* < 0.001) (Fig. 2a). The modified log-rank test and CICR survival method analysis revealed a significantly higher incidence of upper and lower GI bleeding in SSc patients than in controls (*p* < 0.001; Fig. 2b and c).

The multivariate-adjusted subdistribution hazards model revealed that SSc was independently associated with over-all GI bleeding risks (sHR 2.98; 95% CI, 2.21–4.02; *p* < 0.001) after adjustments for age, sex, comorbidities, concomitant medication, and time-dependent covariates (Table 3). SSc was associated with upper GI bleeding (sHR, 2.80; 95% CI, 1.92–4.08; *p* < 0.001) and lower GI bleeding (sHR, 3.93; 95% CI, 2.52–6.13; *p* < 0.001). Among upper GI bleeding, both peptic ulcer bleeding (sHR, 2.52; 95% CI, 1.64–3.88; *p* < 0.001) and non-peptic ulcer bleeding (sHR, 3.57; 95% CI, 2.27–5.61; *p* < 0.001) were found increased risks in SSc patients (Table 3).

**Table 3:** Risk factors associated with gastrointestinal bleeding among all the enrollees by using univariate and multivariate Cox regression analyses

Variables	Univariate analysis			Multivariate analysis^a^		
	HR	95% CI	*p*	HR	95% CI	*p*
All gastrointestinal bleeding	3.73	2.83–4.92	< 0.001	2.98	2.21–4.02	< 0.001
Upper gastrointestinal bleeding	3.50	2.48–4.94	< 0.001	2.80	1.92–4.08	< 0.001
Peptic ulcer bleeding	3.03	2.03–4.53	< 0.001	2.52	1.64–3.88	< 0.001
Non-peptic ulcer bleeding	4.79	3.16–7.27	< 0.001	3.57	2.27–5.61	< 0.001
Lower gastrointestinal bleeding	5.14	3.40–7.77	< 0.001	3.93	2.52–6.13	< 0.001

*Abbreviations*: *HR* hazard ratio, *CI* confidence interval

^a^Each variable was adjusted for age, sex, comorbidities, concomitant medications, competing mortality, and time-dependent covariate

Male gender (sHR, 1.52; 95% CI, 1.31–1.76; *p <* 0.001), increased age (sHR, 1.05; 95% CI, 1.05–1.06; *p <* 0.001), comorbidities with diabetes (sHR, 1.46; 95% CI, 1.19–1.79; *p <* 0.001) or hypertension (sHR, 1.38; 95% CI, 1.15–1.65; *p <* 0.001) were all related to higher over-all GI bleeding risks (Additional file 1: Table S3). In addition, NSAIDs were found associated with over-all GI bleeding risk (sHR, 1.23; 95% CI, 1.05–1.44; *p =* 0.009), upper GI bleeding risk (sHR, 1.31; 95% CI, 1.08–1.58; *p =* 0.005), and peptic ulcer bleeding risk (sHR, 1.37; 95% CI, 1.12–1.68; *p =* 0.003) in multivariate analysis model (Additional file 1: Table S3–S5). Antiplatelet demonstrated higher non-peptic ulcer bleeding risks (sHR, 1.45; 95% CI, 1.08–1.95; *p =* 0.015) and lower GI bleeding risks (sHR, 1.42; 95% CI, 1.05–1.91; *p =* 0.021) (Additional file 1: Table S6, S7). Steroid was also related to higher non-peptic ulcer bleeding risks (sHR, 1.49; 95% CI, 1.07–2.06; *p =* 0.017) and lower GI bleeding risks (sHR, 1.55; 95% CI, 1.13–2.14; *p =* 0.007) in the present study (Additional file 1: Table S6, S7).

### Multivariate stratified analyses for associations between SSc and GI bleeding

The association between SSc and GI bleeding was further analyzed using the subdistribution hazards model stratified by baseline risk factors for GI bleeding, with adjustments for age, sex, confounders, and time-dependent variables. In each stratum, SSc was significantly associated with a higher risk of over-all GI bleeding risks except for the stratum by patients with CKD (Additional file 1: Figure S1). A similar increased risk pattern was observed for upper GI bleeding, lower GI bleeding, and peptic ulcer bleeding risks (Additional file 1: Figure S2, S3, S5). In the stratified analysis of non-peptic ulcer bleeding, SSc patients showed higher bleeding risks in all strata (Additional file 1: Figure S4).

## Discussion

### Principal observations

The relationship of SSc with GI bleeding risk was known but the exact risk ratios had not been well established previously. Using a large-scale nationwide database, the over-all GI bleeding risk, either upper GI bleeding or lower GI bleeding, was increased in SSc patients compared to non-SSc controls. Moreover, both peptic ulcer bleeding or non-peptic ulcer bleeding risks were increased in SSc patients. Further multivariate stratified analyses also validated the results. Several well-known GI bleeding risks related to drugs (NSAIDs, antiplatelets, steroids) were also found associated with over-all GI bleeding events and events in different GI bleeding subtypes.

### Possible underlying mechanisms

SSc could cause GI mucosal abnormalities, thereby contributing to GI bleeding, including that of the upper and lower GI tract, through several mechanisms. SSc is characterized by autoimmunity, inflammation, and small-vessel vasculopathy, which may contribute to GI bleeding events. Current evidence presented the association between SSc and GI mucosal telangiectasia (as part of the CREST syndrome) which is considered the most common source of bleeding in SSc patients. Moreover, gastric antral venous ectasia, rare but related to SSc, is the other possible etiology of upper GI bleeding in SSc patients [8, 21, 22]. Therefore, SSc patients have a risk of GI vascular mucosal abnormalities. Additionally, mucosal inflammation of the esophagus, stomach, and duodenum could be observed through esophagogastroduodenoscopy [23]. Clinical symptoms of GI involvement are significantly more common in patients with diffuse cutaneous SSc [23]. In approximately 10% of SSc patients, GI disease develops before cutaneous manifestations occur [24]. The esophagus is the most frequently affected [24–26] organ, followed by the anorectum, stomach, small bowel, and colon [23, 27, 28]. Therefore, the incidence of upper GI symptoms is significantly higher than that of lower GI symptoms [23]. As shown in our reports, the incidence rate of GI bleeding was higher in the upper GI tract than in the lower GI tract. Thus, non-peptic ulcer bleeding is more common than peptic ulcer bleeding.

### Comparison with previous studies

In a hospital-based study of 144 patients, 15% of SSc patients had more than one episode of GI bleeding. The most common cause was mucosal telangiectasias (41%), followed by peptic ulcer disease (32%) and erosive gastritis. Regarding the sex difference, a cross-sectional study showed that a higher risk of severe GI manifestations was found in men, which was consistent with our results [29]. Studies have also suggested that patients with SSc/CREST syndrome have a risk of severe GI involvement at an early stage (< 2 years from onset) [8, 30]. The present results also demonstrated an increased GI bleeding risk with increasing age in SSc patients.

### Clinical impact and application

This study presented several clinical implications. The comorbidities and complications difference between SSc patients and controls seem to be growing with time. SSc patients were known to be related to vascular complications [31]. In addition, physicians caring for SSc patients also need to focus on the higher risks of over-all GI bleeding, particularly in male patients, comorbidities with diabetes mellitus or hypertension, and avoid prescription drugs with potential GI bleeding side effects. For example, pain related to peripheral insufficiency is exquisite, so treatment of the pain by NSAIDs or improvement of peripheral circulation by antiplatelet should be instituted promptly and adequately escalated. Adequate GI bleeding risk assessment may ensure that preventive measures are promptly taken. Among higher GI bleeding risks patients (male gender, elderly, diabetes, and hypertension), NSAIDs should be minimized or avoided in favor of acetaminophen or opiates since NSAIDs have a risk of GI bleeding risks. Moreover, physicians should make efforts to improve bowel motility and the nutritional status of SSc patients, limit steroids use, and attempt to reduce non-peptic ulcer or lower GI bleeding risk in these patients.

### Strengths and limitations

The strength of our study was the use of a population-based data set with a large sample size, which enabled us to trace the differences between the two groups. Furthermore, the certification of SSc as a catastrophic illness is strict and exempts patients from related medical expenses in the health care system. Some limitations must still be addressed. First, this was a retrospective cohort study based on diagnostic codes and prescription history. Data on some crucial clinical features (such as disease duration, the distinction of localized, limited, or diffuse SSc) and laboratory parameters (such as autoantibody profile) were not recorded in the NHIRD; therefore, we could not evaluate SSc severity. SSc diagnoses were not made based on the 2013 ACR/EULAR (European League Against Rheumatism) classification criteria, thus limiting the generalizability of the results to patients with earlier or milder SSc who only fulfilled the new classification criteria [32, 33]. Second, our definition of GI bleeding ensured that only events sufficiently severe to warrant hospitalization were included. Thus, the estimates of bleeding risk could be considered conservative and did not include obscure bleeding events not requiring hospitalization. The higher threshold at admission reflects the significantly lower incidences of GI bleeding events in SSc patients. In addition, misclassified coding may have occurred when most individuals with obscure GI bleeding did not undergo capsule endoscopy or enteroscopy to explore the bleeding source [12, 13, 15]. Miscoding could not be completely avoided in this epidemiological study, but the misclassification was likely to be nondifferentiated between the two groups. Nevertheless, considering the high accessibility of patients to health care in Taiwan and considering that patients who do not seek medical advice usually have milder or no symptoms, this bias may be of little clinical importance. Furthermore, the separated outcome of angiodysplasia in SSc patients was not investigated in the present study because of the diagnosis coding of angiodysplasia was not well-validated in Taiwan NHIRD. Third, being an administrative data set, the NHIRD did not provide personal information, such as smoking, alcohol consumption, and nutritional status. To minimize these confounding factors, the major medical comorbid diseases were assessed between SSc patients and non-SSc controls; however, this may not have allowed for adequate adjustment for unmeasured confounders. By using joint modeling of multiple diseases [34], we captured the effect of smoking by investigating its proxies, namely COPD and coronary artery disease, which are strongly correlated with smoking. Moreover, SSc was found to be associated with GI bleeding independent of the effects of these proxies. Forth, the over-the-counter usage of NSAID was not able to calculate, and this might be the reason why the proportion of NSAID usage was low in this present study. Lastly, patients with SSc have more comorbidities compare to matched cohort. The surveillance bias cannot be excluded because comorbidities are more likely to be detected in the case group. However, the accessibility to medical service is high in Taiwan so GI discomfort or bleeds should be equally reported in both groups.

## Conclusion

SSc patients independently exhibit increased GI bleeding risk, including that of the upper (peptic and non-peptic ulcer) and lower GI bleeding, particularly in men and those with diabetes mellitus, hypertension, and adverse drug exposure (NSAIDs, antiplatelets, steroids). Gastroenterologists, rheumatologists, and primary health care providers should be aware of GI bleeding risk in SSc patients, and esophagogastroduodenoscopy and monitoring for GI complications should be considered.

## Supplementary information

**Additional file 1 : Table S1.** Corresponding ICD-9 codes for the diagnoses of diseases examined in this study. **Table S2.** Drug (Anatomical Therapeutic Chemical code) concomitant prescriptions in the present study. **Table S3.** Cox proportional hazards regression model analysis for risk of over-all gastrointestinal bleeding. **Table S4.** Cox proportional hazards regression model analysis for the risk of upper gastrointestinal bleeding. **Table S5.** Cox proportional hazards regression model analysis for risk of peptic ulcer gastrointestinal bleeding. **Table S6.** Cox proportional hazards regression model analysis for risk of non-peptic ulcer gastrointestinal bleeding. **Table S7.** Cox proportional hazards regression model analysis for risk of lower gastrointestinal bleeding. **Figure S1.** Multivariate-adjusted analysis of over-all gastrointestinal bleeding risks stratified by age, sex, diabetes mellitus, hypertension, hyperlipidemia, coronary artery disease (CAD), chronic obstructive pulmonary disease (COPD), and chronic kidney disease (CKD). **Figure S2.** Multivariate-adjusted analysis of upper gastrointestinal bleeding risks stratified by age, sex, diabetes mellitus, hypertension, hyperlipidemia, coronary artery disease (CAD), chronic obstructive pulmonary disease (COPD), and chronic kidney disease (CKD). **Figure S3.** Multivariate-adjusted analysis of peptic ulcer gastrointestinal bleeding risks stratified by age, sex, diabetes mellitus, hypertension, hyperlipidemia, coronary artery disease (CAD), chronic obstructive pulmonary disease (COPD), and chronic kidney disease (CKD). **Figure S4.** Multivariate-adjusted analysis of non-peptic ulcer gastrointestinal bleeding risks stratified by age, sex, diabetes mellitus, hypertension, hyperlipidemia, coronary artery disease (CAD), chronic obstructive pulmonary disease (COPD), and chronic kidney disease (CKD). **Figure S5.** Multivariate-adjusted analysis of lower gastrointestinal bleeding risks stratified by age, sex, diabetes mellitus, hypertension, hyperlipidemia, coronary artery disease (CAD), chronic obstructive pulmonary disease (COPD), and chronic kidney disease (CKD).

## Data Availability

The data that support the findings of this study are available from the National Health Insurance Research Database (NHRID), but restrictions apply to the availability of these data, which were used under license for the present study and so are not publicly available. Data are however available from the authors upon reasonable request and with permission of NHRID.

## Abbreviations

GI: Gastrointestinal
SSc: Systemic sclerosis
COPD: Chronic obstructive pulmonary disease
PPI: Proton pump inhibitor
H2RA: Histamine type 2 receptor antagonist
NSAID: Nonsteroidal anti-inflammatory drugs
COX-2: Cyclooxygenase-2
CI: Confidence interval
NHIRD: National Health Insurance Research Database
NHI: National Health Insurance
ICD-9: International Classification of Disease, Ninth Revision
CICR: Cumulative incidence competing risk

## References

[1] Volkmann ER, Varga J (2019). Emerging targets of disease-modifying therapy for systemic sclerosis. Nat Rev Rheumatol.

[2] Asano Y (2018). Systemic sclerosis. J Dermatol.

[3] Kuo CF, See LC, Yu KH, Chou IJ, Tseng WY, Chang HC (2011). Epidemiology and mortality of systemic sclerosis: a nationwide population study in Taiwan. Scand J Rheumatol.

[4] Rosa JE, Soriano ER, Narvaez-Ponce L, del Cid CC, Imamura PM, Catoggio LJ (2011). Incidence and prevalence of systemic sclerosis in a healthcare plan in Buenos Aires. J Clin Rheumatol.

[5] Ranque B, Mouthon L (2010). Geoepidemiology of systemic sclerosis. Autoimmun Rev.

[6] Tyndall AJ, Bannert B, Vonk M, Airo P, Cozzi F, Carreira PE (2010). Causes and risk factors for death in systemic sclerosis: a study from the EULAR Scleroderma Trials and Research (EUSTAR) database. Ann Rheum Dis.

[7] Marie I, Antonietti M, Houivet E, Hachulla E, Maunoury V, Bienvenu B (2014). Gastrointestinal mucosal abnormalities using videocapsule endoscopy in systemic sclerosis. Aliment Pharmacol Ther.

[8] Duchini A, Sessoms SL (1998). Gastrointestinal hemorrhage in patients with systemic sclerosis and CREST syndrome. Am J Gastroenterol.

[9] Lin LY, Warren-Gash C, Smeeth L, Chen PC (2018). Data resource profile: the National Health Insurance Research Database (NHIRD). Epidemiol Health.

[10] Hsieh CY, Su CC, Shao SC, Sung SF, Lin SJ, Kao Yang YH (2019). Taiwan’s National Health Insurance Research Database: past and future. Clin Epidemiol.

[11] Preliminary criteria for the classification of systemic sclerosis (scleroderma) (1980). Subcommittee for scleroderma criteria of the American Rheumatism Association Diagnostic and Therapeutic Criteria Committee. Arthritis Rheum.

[12] Peng YL, Hu HY, Luo JC, Hou MC, Lin HC, Lee FY (2014). Alendronate, a bisphosphonate, increased upper and lower gastrointestinal bleeding: risk factor analysis from a nationwide population-based study. Osteoporosis Int.

[13] Lin CC, Hu HY, Luo JC, Peng YL, Hou MC, Lin HC (2013). Risk factors of gastrointestinal bleeding in clopidogrel users: a nationwide population-based study. Aliment Pharmacol Ther.

[14] Luo JC, Leu HB, Huang KW, Huang CC, Hou MC, Lin HC (2011). Incidence of bleeding from gastroduodenal ulcers in patients with end-stage renal disease receiving hemodialysis. CMAJ.

[15] Luo JC, Leu HB, Hou MC, Huang KW, Lin HC, Lee FY (2013). Nonpeptic ulcer, nonvariceal gastrointestinal bleeding in hemodialysis patients. Am J Med.

[16] Shiao TH, Liu CJ, Luo JC, Su KC, Chen YM, Chen TJ (2013). Sleep apnea and risk of peptic ulcer bleeding: a nationwide population-based study. Am J Med.

[17] Hsu YC, Lin JT, Chen TT, Wu MS, Wu CY (2012). Long-term risk of recurrent peptic ulcer bleeding in patients with liver cirrhosis: a 10-year nationwide cohort study. Hepatology..

[18] Gray RJ (1988). A class of K -sample tests for comparing the cumulative incidence of a competing risk. Ann Stat.

[19] Austin PC, Lee DS, Fine JP (2016). Introduction to the analysis of survival data in the presence of competing risks. Circulation..

[20] Jason P, Fine RJG (1999). A proportional hazards model for the subdistribution of a competing risk. J Am Stat Assoc.

[21] Plastiras SC, Tzivras M, Vlachoyiannopoulos PG (2007). Severe gastrointestinal involvement in systemic sclerosis. Clin Rheumatol.

[22] Ebert EC (2008). Gastric and enteric involvement in progressive systemic sclerosis. J Clin Gastroenterol.

[23] Wielosz E, Borys O, Zychowska I, Majdan M (2010). Gastrointestinal involvement in patients with systemic sclerosis. Polskie Archiwum Medycyny Wewnetrznej.

[24] Ebert EC (2006). Esophageal disease in scleroderma. J Clin Gastroenterol.

[25] Szamosi S, Szekanecz Z, Szucs G (2006). Gastrointestinal manifestations in Hungarian scleroderma patients. Rheumatol Int.

[26] Ntoumazios SK, Voulgari PV, Potsis K, Koutis E, Tsifetaki N, Assimakopoulos DA (2006). Esophageal involvement in scleroderma: gastroesophageal reflux, the common problem. Semin Arthritis Rheum.

[27] Marie I, Levesque H, Ducrotte P, Denis P, Hellot MF, Benichou J (2001). Gastric involvement in systemic sclerosis: a prospective study. Am J Gastroenterol.

[28] McFarlane IM, Bhamra MS, Kreps A, Iqbal S, Al-Ani F, Saladini-Aponte C, et al. Gastrointestinal manifestations of systemic sclerosis. Rheumatology (Sunnyvale). 2018;8(1). 10.4172/2161-1149.1000235. Epub 2018 Mar 30. 10.4172/2161-1149.1000235 PMC6059963 30057856

[29] McMahan ZH, Paik JJ, Wigley FM, Hummers LK (2018). Determining the risk factors and clinical features associated with severe gastrointestinal dysmotility in systemic sclerosis. Arthritis Care Res.

[30] Nishimagi E, Tochimoto A, Kawaguchi Y, Satoh T, Kuwana M, Takagi K (2007). Characteristics of patients with early systemic sclerosis and severe gastrointestinal tract involvement. J Rheumatol.

[31] Elhai M, Avouac J, Walker UA, Matucci-Cerinic M, Riemekasten G, Airo P (2016). A gender gap in primary and secondary heart dysfunctions in systemic sclerosis: a EUSTAR prospective study. Ann Rheum Dis.

[32] van den Hoogen F, Khanna D, Fransen J, Johnson SR, Baron M, Tyndall A (2013). 2013 classification criteria for systemic sclerosis: an American College of Rheumatology/European League against Rheumatism collaborative initiative. Arthritis Rheum.

[33] Giacomelli R, Afeltra A, Alunno A, Baldini C, Bartoloni-Bocci E, Berardicurti O (2017). International consensus: what else can we do to improve diagnosis and therapeutic strategies in patients affected by autoimmune rheumatic diseases (rheumatoid arthritis, spondyloarthritides, systemic sclerosis, systemic lupus erythematosus, antiphospholipid syndrome and Sjogren’s syndrome)?: the unmet needs and the clinical grey zone in autoimmune disease management. Autoimmun Rev.

[34] Best N, Hansell AL (2009). Geographic variations in risk: adjusting for unmeasured confounders through joint modeling of multiple diseases. Epidemiology..

