# Supplementary material for: High risk of gastrointestinal hemorrhage in patients with systemic sclerosis
Source: Arthritis Res Ther. 2019 Dec 26;21:301. doi: 10.1186/s13075-019-2078-5 (PMC6933921; doi:10.1186/s13075-019-2078-5)
Supplement: Supplementary file 1 — Additional file 1 : Table S1. Corresponding ICD-9 codes for the diagnoses of diseases examined in this study. Table S2. Drug (Anatomical Therapeutic Chemical code) concomitant prescriptions in the present study. Table S3. Cox proportional hazards regression model analysis for risk of over-all gastrointestinal bleeding. Table S4. Cox proportional hazards regression model analysis for the risk of upper gastrointestinal bleeding. Table S5. Cox proportional hazards regression model analysis for risk of peptic ulcer gastrointestinal bleeding. Table S6. Cox proportional hazards regression model analysis for risk of non-peptic ulcer gastrointestinal bleeding. Table S7. Cox proportional hazards regression model analysis for risk of lower gastrointestinal bleeding. Figure S1. Multivariate-adjusted analysis of over-all gastrointestinal bleeding risks stratified by age, sex, diabetes mellitus, hypertension, hyperlipidemia, coronary artery disease (CAD), chronic obstructive pulmonary disease (COPD), and chronic kidney disease (CKD). Figure S2. Multivariate-adjusted analysis of upper gastrointestinal bleeding risks stratified by age, sex, diabetes mellitus, hypertension, hyperlipidemia, coronary artery disease (CAD), chronic obstructive pulmonary disease (COPD), and chronic kidney disease (CKD). Figure S3. Multivariate-adjusted analysis of peptic ulcer gastrointestinal bleeding risks stratified by age, sex, diabetes mellitus, hypertension, hyperlipidemia, coronary artery disease (CAD), chronic obstructive pulmonary disease (COPD), and chronic kidney disease (CKD). Figure S4. Multivariate-adjusted analysis of non-peptic ulcer gastrointestinal bleeding risks stratified by age, sex, diabetes mellitus, hypertension, hyperlipidemia, coronary artery disease (CAD), chronic obstructive pulmonary disease (COPD), and chronic kidney disease (CKD). Figure S5. Multivariate-adjusted analysis of lower gastrointestinal bleeding risks stratified by age, sex, diabetes mellitus, hypertension, hype [file 13075_2019_2078_MOESM1_ESM.docx]

**Table S1.** Clinical characteristics of systemic sclerosis patients and age- and sex-matched controls

|  | Case (n=3,665) | Control (n=18,325) |  |
| --- | --- | --- | --- |
|  | N (%) | N (%) | p value |
| **Age, years** | 49.1 ±14.9 | 49.1 ± 14.9 | 1 |
| **Sex** |  |  |  |
| Male | 760 ( 20.7 ) | 3,800 ( 20.7 ) | 1 |
| **Comorbidity** |  |  |  |
| Diabetes mellitus | 374 ( 10.4 ) | 1,090 ( 5.9 ) | <0.001 |
| Hypertension | 723 ( 20.0 ) | 2,116 ( 11.5 ) | <0.001 |
| Dyslipidemia | 424 ( 11.7 ) | 829 ( 4.5 ) | <0.001 |
| Coronary artery disease | 339 ( 9.4 ) | 770 ( 4.2 ) | <0.001 |
| Chronic kidney disease | 121 ( 3.3 ) | 116 ( 0.6 ) | <0.001 |
| Chronic obstructive pulmonary disease | 461 ( 12.8 ) | 916 ( 5.0 ) | <0.001 |
| History of uncomplicated peptic ulcer disease | 262 ( 7.1 ) | 644 ( 3.5 ) | <0.001 |
| **Medications** |  |  |  |
| Antiplatelets | 491 ( 13.4 ) | 705 ( 3.8 ) | <0.001 |
| Warfarin | 45 ( 1.2 ) | 43 ( 0.2 ) | <0.001 |
| Traditional nonsteroidal anti-inflammatory drugs | 1,250 ( 34.1 ) | 3,015 ( 16.5 ) | <0.001 |
| Cyclooxygenase-2 inhibitors | 485 ( 13.2 ) | 100 ( 0.5 ) | <0.001 |
| Proton pump inhibitors | 155 ( 4.2 ) | 103 ( 0.6 ) | <0.001 |
| Histamine type 2 receptor antagonist | 101 ( 2.8 ) | 233 ( 1.3 ) | <0.001 |
| Steroids | 1370 ( 37.4 ) | 564 ( 3.1 ) | <0.001 |

**Table S2.** Incident rates of gastrointestinal bleeding among systemic sclerosis and control groups

|  | Systemic sclerosis (n=3,665) | | Controls (n=18,325) | |  |
| --- | --- | --- | --- | --- | --- |
| Clinical Outcome | No. | Incidence Rate^a^  (95% CI) | No. | Incidence Rate^a^  (95% CI) | Incidence Rate Ratio (95% CI) |
| All gastrointestinal bleeding | 191 | 976.37 (847.27-1125.137) | 675 | 410.76 (380.91-442.95) | 2.38 (2.02-2.79) |
| Upper gastrointestinal bleeding | 117 | 591.75 (493.68-709.30) | 473 | 286.89 (262.17-313.94) | 2.06 (1.68-2.53) |
| Peptic ulcer bleeding | 84 | 422.96 (341.53-523.81) | 393 | 238.01 (215.60-262.74) | 1.78 (1.40-2.25) |
| Non-peptic ulcer bleeding | 101 | 507.83 (417.85-617.19) | 281 | 169.46 (150.76-190.48) | 3.00 (2.39-3.76) |
| Lower gastrointestinal bleeding | 105 | 528.20 (436.24-639.53) | 277 | 167.06 (148.50-187.94) | 3.16 (2.53-3.96) |

^a^ Incidence of gastrointestinal bleeding: per 100,000 person-years.

**Table S3.** Risk factors associated with gastrointestinal bleeding among all the enrollees by using univariate and multivariate Cox regression analyses

| Variables | Univariate analysis | | | Multivariate analysis^a^ | | |
| --- | --- | --- | --- | --- | --- | --- |
|  | HR | 95% CI | *p* | HR | 95% CI | *p* |
| All gastrointestinal bleeding | 3.73 | 2.83-4.92 | <0.001 | 2.98 | 2.21-4.02 | <0.001 |
| Upper gastrointestinal bleeding | 3.50 | 2.48-4.94 | <0.001 | 2.80 | 1.92-4.08 | <0.001 |
| Peptic ulcer bleeding | 3.03 | 2.03-4.53 | <0.001 | 2.52 | 1.64-3.88 | <0.001 |
| Non-peptic ulcer bleeding | 4.79 | 3.16-7.27 | <0.001 | 3.57 | 2.27-5.61 | <0.001 |
| Lower gastrointestinal bleeding | 5.14 | 3.40-7.77 | <0.001 | 3.93 | 2.52-6.13 | <0.001 |

Abbreviations: HR, hazard ratio; CI, confidence interval

^a^Each variable was adjusted for age, sex, comorbidities, concomitant medications, competing mortality, and time-dependent covariate
